# The cryobiopsy in interstitial lung diseases guided by probe‐based confocal laser endomicroscopy is feasible

**DOI:** 10.1111/crj.13669

**Published:** 2023-08-16

**Authors:** Yu Zheng, Liyan Zhang, Yueyan Lou, Bijun Fan, Yongqi Cui, Xueling Wu, Xiaoming Tan

**Affiliations:** ^1^ Department of Respiratory Medicine, Ren Ji Hospital Shanghai Jiao Tong University School of Medicine Shanghai China

**Keywords:** interstitial lung diseases, probe‐based confocal laser endomicroscopy, real‐time imaging, transbrochial lung cryobiopsy

## Abstract

**Background:**

Transbronchial lung cryobiopsy (TBLB) is routinely used to diagnose the interstitial lung disease (ILD). These results are consistent with those of surgical lung biopsy. Fluoroscopy is also used to confirm the final position of the cryoprobe; however, it can increase radiation exposure for both patients and medical care personnel. Probe‐based confocal laser endomicroscopy (pCLE) is a novel optical imaging technique that allows real‐time imaging at the cellular level in vivo. pCLE technology can also be used to identify malignancy, acute rejection in lung transplantation, amiodarone lung, and pulmonary alveolar proteinosis and visualize elastin fibres in the alveolar compartment.

**Objectives:**

The aim of this study is to investigate the ability of pCLE to distinguish fibrotic pulmonary issues from normal lung disease and the safety and feasibility of CLE‐guided bronchoscopy and transbronchial lung cryobiopsy (TBLC) in patients with interstitial lung disease (ILD).

**Methods:**

pCLE images from 17 ILD patients were obtained during TBLB. These images were then compared with histology results to assess the correspondence rate.

**Results:**

pCLE imaging of the alveolar structures was performed. Key characteristics were visible, which could potentially influence the diagnostic rate (fibrotic areas) and the complication rate (blood vessel and pleura).

**Conclusion:**

pCLE may reduce complications and increase the diagnostic yield. It is a potential guidance tool for cryobiopsy in the patients with ILD without fluoroscopy.

## Graphical abstract

Probe‐based confocal laser endomicroscopy (pCLE) may reduce complications and increase diagnostic yield. Thus, it can be a potential guidance tool for cryobiopsy in patients with ILD without fluoroscopy.

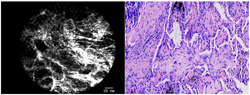

## 1 INTRODUCTION

Transbronchial lung cryobiopsy (TBLC) is a novel technique of lung tissue sampling for the diagnosis of interstitial lung disease (ILD)^1^ and has high degree consistency with surgical lung biopsy (SLB) diagnosis that determines the important role of TBLC in the clinical utility of interstitial lung disease.^2^ However, as reported in previous studies, this technique has two major limitations. First, the diagnostic yield of TBLC for diffuse lung disease was 70%–80%,^3^, ^4^ and TBLB has higher complications (bleeding and pneumothorax) than the forceps biopsy.^1^, ^5^, ^6^

Recently, an “Expert Statement” from the Cryobiopsy Working Group has sought to provide some clear suggestions for the optimum sampling technique.^7^ The working group highlighted that biopsies within 1 cm from the pleura were significantly associated with an increased risk of pneumothorax.^8^ In contrast, biopsies taken approximately from the middle third of the lung increase the probability of severe bleeding because there are medium‐sized arteries in this region that accompany with the bronchus.^9^ Up to now, pre‐procedure high‐resolution CT localization before the bronchoscope is generally recommended. The cryoprobe is inserted until it touches the visceral pleura gently and then withdrawn 1–2 cm to a final position equating to approximately 1 cm from the pleura for biopsy.^8^, ^9^, ^10^, ^11^ In addition, several clinicians use fluoroscopy to determine the final position of the cryoprobe,^12^ but this procedure may lead to radiation exposure for patients and medical care personnel if performed frequently and consumes additional space, manpower, and costs (install a shield room).

In recent years, probe‐based confocal laser endomicroscopy (pCLE) is an emerging optical imaging technology (488 nm) that can observe real‐time imaging at the cellular level in vivo.^13^ In pilot studies, pCLE techniques have already been used to diagnose malignancy, acute rejection in lung transplantation, amiodarone lung, pulmonary alveolar proteinosis, and elastin fibres visible in alveolar chambers.^14^, ^15^, ^16^, ^17^ Furthermore, the pCLE microprobe with an outer diameter of 1.4 mm enables it to reach the (sub)pleural region of the lung, which can distinguish fibrotic pulmonary tissue from normal lung tissue in vivo.^17^ This provides an opportunity for the pCLE technology to be used as a guidance tool for TBLC without fluoroscopy.

The preliminary aim of this study was to evaluate the feasibility and safety of only pCLE‐guided transbronchial lung cryobiopsy (TBLC) in patients with interstitial lung disease (ILD).

## 2 MATERIALS AND METHODS

This study was a prospective, observational, single‐centre clinical trial. Non‐smoking (for at least 3 months) patients with ILD confirmed by high‐resolution enhanced CT scan requiring histological examination were selected for inclusion in this study between October 2018 and April 2020. All patients were aged between 18 and 80 years old. Written informed consent was obtained from all participants.

Exclusion criteria included the following: severe hypertension or arrhythmia; recent myocardial infarction or a history of unstable angina; severe heart, lung, liver, or kidney dysfunction, or extreme systemic failure; severe pulmonary hypertension (systolic pulmonary artery pressure > 50 mmHg assessed by echocardiography); severe superior vena cava syndrome; coagulopathy (international normalized ratio >  1.3, partial thromboplastin time above normal range) or severe thrombocytopenia (absolute platelet count < 50 × 10^9^/L) that cannot be corrected after anticoagulant therapy; ILD in acute aggravated phase; severe impaired lung function (FVC < 50% predicted and D_L_CO < 35% predicted); hypoxaemia while breathing room air (SpO_2_ < 90%), diffusing capacity for carbon monoxide less than 40% predicted.

A flexible bronchoscope was used for examination. The procedure was performed under general anaesthesia or deep sedation, and intubation with controlled mechanical ventilation established used oral/nasal tracheal tube of 8.0 mm diameter. Patient monitoring included continuous oxygen saturation, ECG monitoring, and repeated non‐invasive BP monitoring. After bronchoscopy was introduced, standard bronchoalveolar lavage (BAL) was performed on the other lobe of the ipsilateral lung to reduce interference with pCLE images.

**FIGURE 1:**
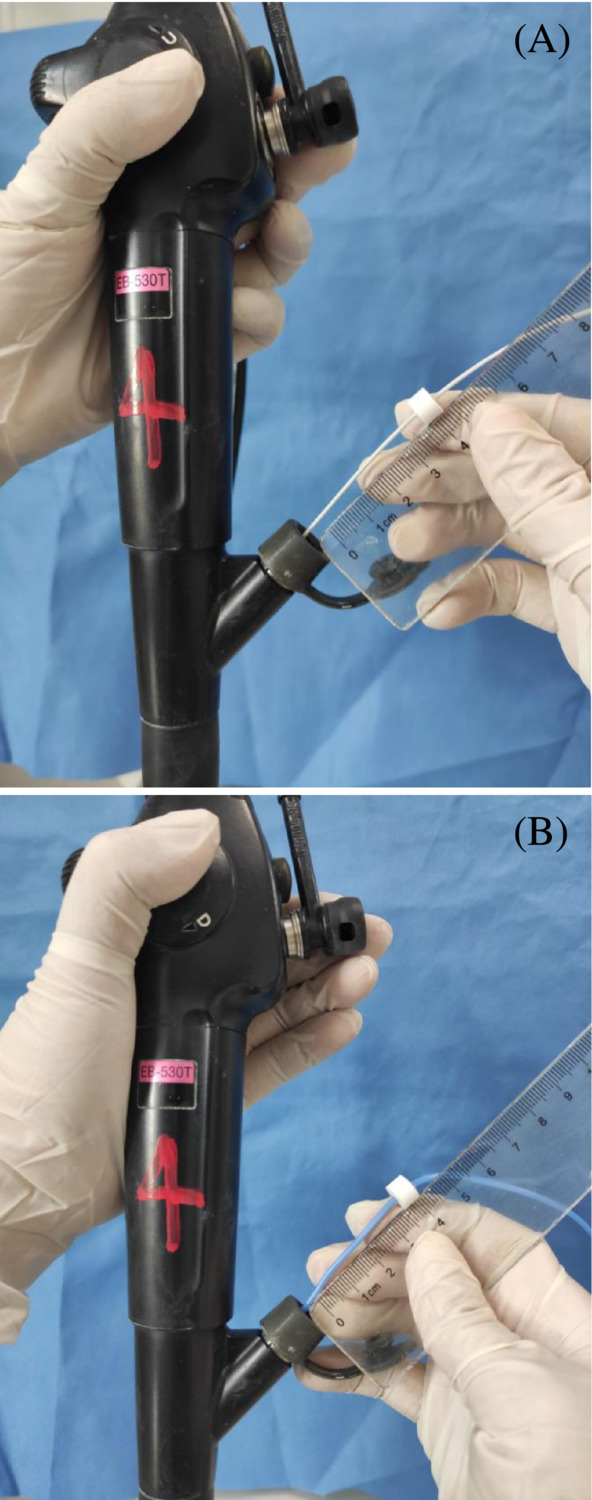
(A) The distance from the target position to the secondary carina was be recorded by marking ring. (B) Marking ring was regressed according to the distance measured before the probe reached the secondary carina.

pCLE probe (AlveoFlex miniprobe [Mauna Kea Technologies, Paris, France] 488‐nm wavelength, resolution 3.5 μm, depth 0–50 μm, field of view 600 μm) was introduced into the working channel of the bronchoscope and slowly proceeding to the alveolar compartment until the pleura was reached. The distance from the secondary carina to the target position was recorded by a marking ring (show in Figure 1A). Several lung segments were assessed in each patient. Images were digitally processed using Cellvizio Viewer software (Mauna Kea Technologies, Paris, France).

The pCLE images were compared with histopathological data obtained during the same endoscopic procedure.

The biopsy locations were previously determined by high‐resolution enhanced CT scan. Cryobiopsy was performed using cryoprobe of 1.9 mm diameter (Erbe Elektromedizin GmbH, Tübingen, Germany) and a freezing time of 3 to 6 s.^7^ A prophylactically placed Fogarty balloon (Arndt Endobronchial Blocker Set, Bloomington, USA) in the segmental lobar bronchus to control bleeding. The cryoprobe was banded and introduced into working channel. The marking ring was regressed according to the distance measured before the probe reached the secondary carina (show in Figure 1B). Then, probe was slowly advanced into target segmental bronchus until its limit and then withdrawn to the marking position. The procedure was repeated two to four times in different areas of the same lobe and guided by pCLE each time.^18^

The samples were fixed in 10% buffered formalin, embedded in paraffin and then sliced into 4‐μm‐thick sections for Elastica van Gieson staining. Histology images of the lung biopsies were assessed by an experienced pulmonary pathologist. A final diagnosis was determined based on patient history, laboratory results, high‐resolution CT scan, BAL, and histology from the lung biopsies after an ILD multidisciplinary discussion session.

## 3 RESULTS

High‐resolution computed tomography (HRCT), pCLE imaging, and TBLC were collected in 17 ILD patients. Clinical characteristics and final diagnosis of the patients are show in Table 1. PCLE imaging of the alveolar compartment was technically feasible in all patients by pCLE imaging (*n* = 17). Sixteen patients were confirmed definitely diagnosed with ILD, including 13 nonspecific interstitial pneumonia (NSIP), one chronic hypersensitivity pneumonitis, one bronchiolitis obliterans organizing pneumonia (BOOP), and one pneumoconiosis. One patient had an inconclusive pathologic diagnosis. The diagnosis rate reached 94.1%. The complication rate was 17.6% (one case of moderate bleeding and two cases of pneumothorax without chest tube).

**TABLE 1:** Clinical characteristics of the patients.

	*N* = 17
Patients	17
Sex (male/female)	10/7
Age, years	50.6 ± 18
TBCB	17
Final diagnosis	16 (94.1%)
Fibrotic NSIP	10
Chronic hypersensitivity pneumonitis	1
Cellular NSIP	3
BOOP	1
Pneumoconiosis	1
Inconclusive final diagnosis	1
Complication of TBCB	3 (17.6%)
Moderate bleeding	1
Pneumothorax (recovery after conservative treatment)	2

*Note*: Values are *n* (%) or mean ± SD, as appropriate. All pneumothorax was recorded. Only moderate (e.g., requiring endoscopic procedures like bronchial occlusion‐collapse and/or instillation of ice‐cold saline) and severe (e.g., causing haemodynamic or respiratory instability, requiring tamponade or other surgical interventions, transfusions, or admission to the intensive care unit) bleeding were recorded for this study.

Abbreviations: BOOP, bronchiolitis obliterans organizing pneumonia; DIP, desquamative interstitial pneumonia; LIP, lymphocytic interstitial pneumonia; NSIP, nonspecific interstitial pneumonia; TBCB, transbronchial cryobiopsy.

**FIGURE 2:**
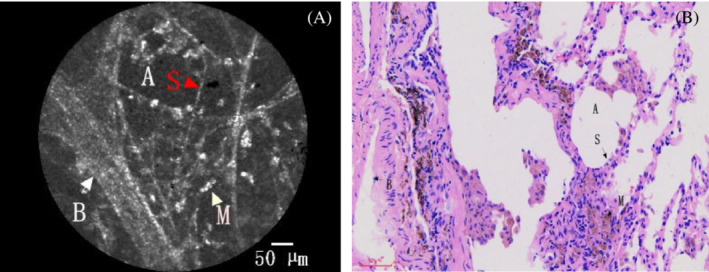
A patient is diagnosed with pneumoconiosis. (A) pCLE imaging visualizes the alveolar airspaces (A) and elastin fibres of the alveolar septum (S). The adjacent blood vessel (B) and macrophages (M) were also seen. (B) Characteristic in vivo pCLE pattern of the alveolar septum showed evenly distributed elastin fibres, compatible with the presence of elastin fibres in the histological HE staining.

**FIGURE 3:**
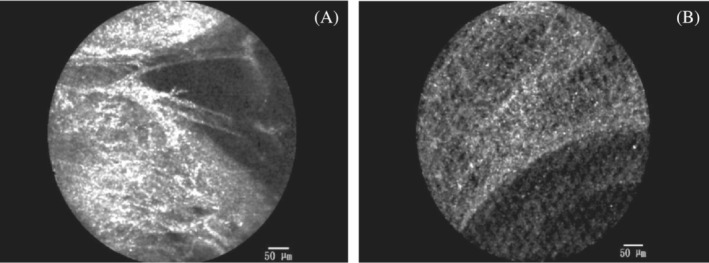
(A) pCLE imaging of the pleura: Dense tissue was observed when probe reached its limit; and (B) bronchial wall: Loose elastin fibres were visible. The density of pleura imaging is higher than bronchial wall.

On the pCLE imaging, the alveolar cavity and the elastin fibres within the alveolar septum could be seen. The adjacent blood vessels were clearly visible (show in Figure 2A). The pleura and bronchial wall could be distinguished based on pCLE imaging (show in Figure 3). A large number of macrophages were observed in pneumoconiosis patients (show in Figure 2A). Characteristic in vivo pCLE pattern of the alveolar septum showed evenly distributed elastin fibres, compatible with the presence of elastin fibres in the histological HE staining (show in Figure 2B).

**FIGURE 4:**
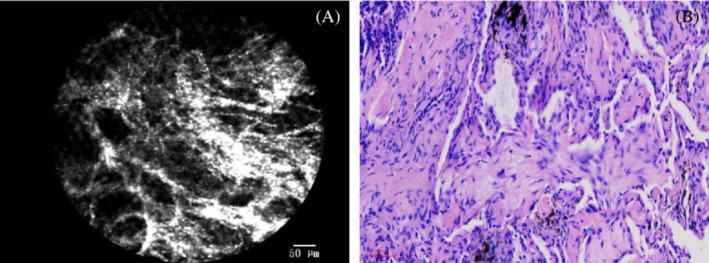
(A) Granulation tissue in alveoli can be seen in the pCLE imaging. (B) Corresponding histology with granulation tissue and Masson body.

We saw granulomatous tissue formation in the alveoli, alveolar ducts, respiratory bronchioles, and the terminal bronchioles of the patient who was diagnosed with BOOP (show in Figure 4A,B).

**FIGURE 5:**
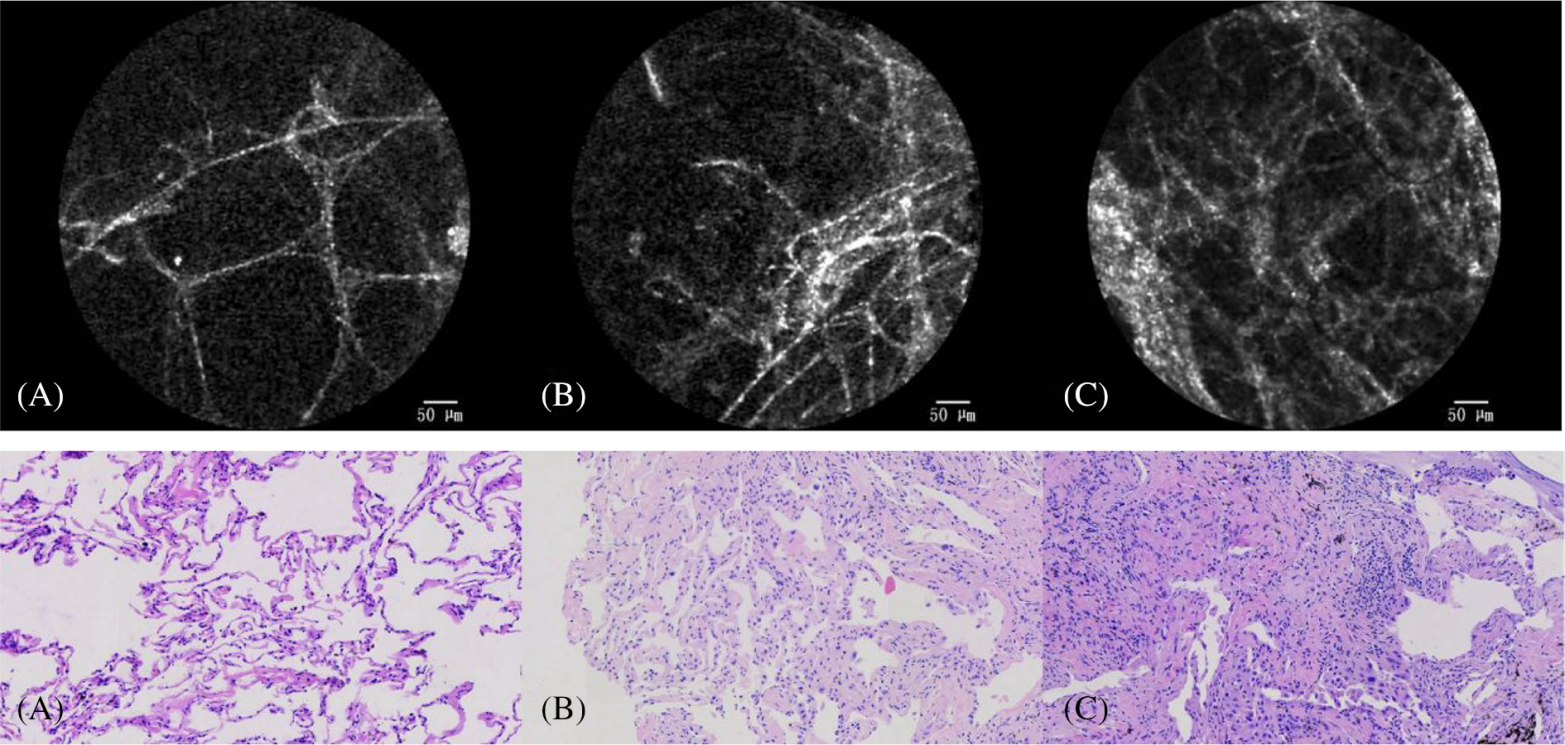
The pCLE imaging (top row) and corresponding histology (bottom row) of a normal pCLE pattern with a thin single‐fibered elastin fibre network and dark alveolar air spaces. (A) Normal alveolar structure without abnormal histology. Mild increase of elastin fibres and preserved alveolar architecture. (B) Mild lung fibrosis areas can be seen. pCLE imaging with destructed alveolar structure and increased elastin fibres. (C) Dense lung fibrosis areas can be seen.

We found that on pCLE imaging, varying degrees of fibrotic lung areas (shown in Figure 5B,C top row) were characterized by a loss of normal (show in Figure 5A top row) alveolar structure and hyperplasia of elastic fibres. These alteration of alveolar structure and increased elastin fibres were consistent with the pattern in the histology of the lung biopsies (show in Figure 5B,C bottom row). The pCLE imaging showed both mild and dense fibrotic lung areas in those patients with pathology‐proven fibrosis. Areas with normal alveoli were alternated by fibrotic lung areas.

**FIGURE 6:**
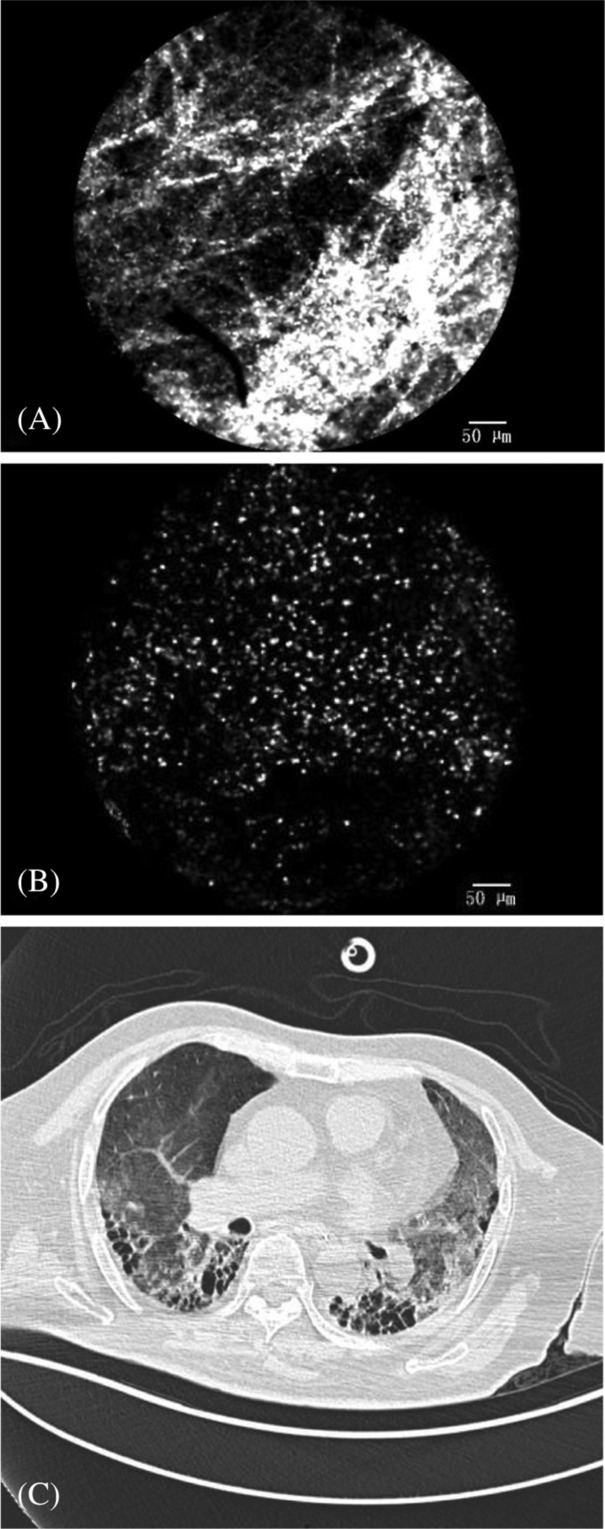
The pCLE (A, densely packed fibres and absent alveolar structures; B, numerous inflammatory cells) and CT images of one suspected dermatomyositis associated diffuse alveolar damage (DAD) patient.

In addition, we performed pCLE technique in one diffuse alveolar damage (DAD) patient due to suspected dermatomyositis (show in Figure 6C). The case was excluded because TBLC was unavailable. Densely packed fibre, absent alveolar structures, and numerous inflammatory cells were observed on pCLE imaging (shown in Figure 6A,B).

## 4 DISCUSSION

ILD is a heterogeneous disease, and diagnosis remains a challenge, requiring integration of clinical and radiological data. Lung biopsy is necessary when there is not enough information to make a diagnosis.

TBLC has advantages in obtaining lung tissue for the diagnosis of ILD. However, the diagnostic guidelines for idiopathic pulmonary fibrosis do not recommend for or against TBLC in their diagnostic algorithms because the diagnostic accuracy has not been verified.^19^ Therefore, finding the interest area and performing biopsy safely is the key of TBLC procedure. Some studies utilized fluoroscopy and/or radial probe endobronchial ultrasound (RP‐EBUS) to locate the final position of the cryoprobe.^12^, ^20^ These procedures may lead to radiation exposure and seem to confirm the hypovascular area to decrease the risk of hemorrhage but could not distinguish areas with lung fibrosis or other abnormalities found in ILD. In addition, fluoroscopy was not available in many bronchoscopy rooms in China.

pCLE has been used to visualize alveolar structure in vivo. Images of normal alveolar compartment show thin cavity that are round, helical, or looped shape. In smoking individuals, highly fluorescent macrophages are visible throughout the alveoli, which has a positive correlation with the number of cigarettes used.^21^ Also, pCLE images of amiodarone induced ILD, pulmonary alveolar microlithiasis, invasive aspergillosis, *Pneumocystis jirovecii* pneumonia, lymphangioleiomyomatosis, and metastatic pulmonary calcification were reported recently.^15^, ^22^, ^23^, ^24^, ^25^, ^26^

Furthermore, several studies have identified specific characteristics of pCLE images in ILD. Meng et al.^27^ identified six different patterns to discriminate chronic fibrosis ILD from other ILDs. Salaün et al.^17^ identified nine specific pCLE characteristics. Based on above, the role of pCLE patterns in ILD diagnosis remains limited, but they were able to discriminate abnormal alveoli structure from normal lung structure.^28^

Therefore, we propose to identify pCLE as the imaging guidance technique of TBLC without fluoroscopy. In this study, we use the probe of pCLE to confirm the distance from the secondary carina to the target position substituting fluoroscopy procedure, in order to avoid the unnecessary exposure. We observed several types of pCLE patterns indicated in previous study^17^ and demonstrated increasing pathology diagnostic yield (94.1% vs. 82.8%^1^) due to that pCLE was capable of identification of bronchial wall, non‐, mild, dense fibrotic, and granulomatous areas in vivo. Our study profiled pCLE technique might reduce the complication rate (17.6% vs. 20.2%^1^) on account of keeping the cryoprobe away from pleura and proximal larger vasculature, and there is potential for pCLE as a high‐resolution guidance tool in vivo.

In our experience, performing the pCLE technique in ILD patients is convenient. We identified the segments of interest through CT scanning and collected pCLE images in a short time. No adverse events related to pCLE technology were observed.

In addition, we performed pCLE technique in one diffuse alveolar damage (DAD) patient due to suspected dermatomyositis in ICU. The case was excluded because TBLC was unavailable. From our observation, we noticed densely packed fibres and alveolar structures absent, which is different of the pattern of acute cardiac failure or infectious pneumonia^15^ (Figure 5A,C). We make an assumption that the specific CLE imaging can distinguish DAD from infectious diseases, but TBLC was not performed by the reason of safety concerning in this case. Despite the following, numerous inflammatory cells were observed in some area, and we suspected sputum blots in small airways (Figure 5B). A recent retrospective study of five patients with ARDS underwent TBLC only by utilizing RP‐EBUS guided. None of them developed pneumothorax. Just two patients developed massive bleeding, which was controlled soon. The complications of TBLC may be acceptable.^29^ It was suggested that this procedure might be recommended in non‐resolving ARDS patient.

To our knowledge, there is only one previous study that assess pCLE in combination with fluoroscopy as a guidance tool for cryobiopsy in ILD.^30^ The current study is the first attempt to investigate TBLC by only using pCLE guide, and the results are coherent.

There are several limitations in this study. First, it was a retrospective study. Second, the results were limited by the small sample size. ILD is a heterogeneous disease; larger prospective studies should be performed to assess in future investigations. In addition, fluoroscopy as a basic radiology measure should be recommend due to the consideration of potential life‐threatening side effects of TBLC especially haemorrhage and tension pneumothorax.

In conclusion, our findings suggest that pCLE is the potential tool to help identify biopsy areas and might reduce complication and improve diagnostic yield. Further investigations with a prospective design and a larger number of patients are required to confirm the value of TBLC guide by pCLE in ILD.

## AUTHOR CONTRIBUTIONS

Yu Zheng and Liyan Zhang contributed equally to this work.

## CONFLICT OF INTEREST STATEMENT

The authors have no conflicts of interest to declare.

## ETHICS STATEMENT

All subjects have given their written informed consent and that the study protocol was approved by the institute's committee of Ren Ji Hospital on human research (2018113).

## Data Availability

The data that support the findings of this study are available upon reasonable request from the authors.
